# Threatened synanthropes depend on intact forests: a critical evaluation of Moore *et al*. (2023)

**DOI:** 10.1111/brv.70007

**Published:** 2025-03-11

**Authors:** Anna Holzner, Nadine Ruppert, Kurnia Ilham, Stefano S. K. Kaburu, André Luiz Koch Liston, Agustin Fuentes, Malene F. Hansen

**Affiliations:** ^1^ The Long‐Tailed Macaque Project Ellepindevej 5 Sorø 4180 Denmark; ^2^ School of Biological Sciences, Universiti Sains Malaysia Minden Penang 11800 Malaysia; ^3^ Malaysian Primatological Society Taman Nuri Kulim Kedah 08000 Malaysia; ^4^ Department of Biology Andalas University Limau Manis, Pauh Padang West Sumatra 25163 Indonesia; ^5^ School of Animal, Rural and Environmental Sciences Nottingham Trent University Brackenhurst Campus Southwell NG25 0QF UK; ^6^ Department of Chemistry Princeton University Washington Road Princeton NJ 08544 USA; ^7^ Department of Chemistry Columbia University 3000 Broadway New York NY 10027 USA; ^8^ Department of Anthropology Princeton University 116 Aaron Burr Hall Princeton NJ 08544 USA; ^9^ Behavioural Ecology Group, Department of Biology University of Copenhagen Universitetsparken 15 Copenhagen 2100 Denmark; ^10^ Oxford Wildlife Trade Research Group, Oxford Brookes University Gipsy Lane Oxford OX3 0BP UK

**Keywords:** *Macaca fascicularis*, *Macaca nemestrina*, relative abundance, *Sus barbatus*, *Sus scrofa*, synanthropes

## Abstract

Synanthropes are known for their remarkable adaptability to coexist with humans, yet increased visibility exposes them to significant threats, such as hunting or conflict over resources. Moore *et al.*'s review ‘The rise of hyperabundant native generalists threatens both humans and nature’ (https://doi.org/10.1111/brv.12985) explores distribution patterns and impacts of macaques and pigs in anthropogenic environments. Our critical evaluation of this study revealed several substantial issues: the pooling of data from species that are ecologically and behaviourally distinct, an error in data acquisition, potential biases in statistical analyses, and critical misrepresentations of threats to and from wildlife in human‐impacted habitats. Additionally, we highlight the lack of evidence supporting the authors' core assertion of hyperabundance of the study species. While Moore *et al.* compare species densities and abundance across various habitat types, their analyses did not demonstrate population increases over time. On the contrary, our re‐analysis of their data sets showed a decreasing population trend in *Macaca nemestrina* and the absence of *M. fascicularis* from 44% of surveyed habitats characterized by medium to high forest integrity. Further, our findings emphasize the importance of intact forests for predicting a high relative abundance of macaques and pigs. Overall, we recommend a more careful interpretation of the data, as misrepresentations of abundance data can result in negative or sensational discourses about overabundance, which may threaten the conservation of species that often thrive in anthropogenic landscapes.

## I. INTRODUCTION

Synanthropes are remarkably adaptable wildlife species that demonstrate a high degree of flexibility in utilizing human resources and thrive in anthropogenic environments, often closely associating with human populations (Fuentes, 2010; Gumert, 2011). Long‐tailed macaques (*Macaca fascicularis*), for example, have coexisted with humans across most parts of their distributional range for millennia (Thierry, 2007b; Fuentes *et al*., 2008; Gumert *et al*., 2011; Hansen *et al*., 2021). Paradoxically, many of these synanthropes find themselves among the world's most threatened species, facing unprecedented risks that challenge their survival (e.g. Luskin *et al*., 2021; Gamalo *et al*., 2023; Holzner *et al*., 2024).

Given the fast rate of global land use change driven by agricultural intensification, expansion of infrastructure, and growing urbanization (McGee, 2001; Winkler *et al*., 2021), investigation aimed at enhancing our understanding of wildlife in anthropogenic landscapes is of great importance. Extrapolating the local population sizes of synanthropes in these landscapes to other areas is a common yet problematic approach (Kyes, Iskandar & Pamugas, 2011; Hansen *et al*., 2019). It carries the risk of creating a misleading image of overabundance, which can shape negative public perceptions and misinform management plans, such as culling or translocating locally abundant populations, potentially threatening the long‐term survival of these species (Hansen *et al*., 2019).

Here, we review Moore *et al*. (2023)'s examination of the distribution patterns and impacts of four generalist species – long‐tailed macaques, southern pig‐tailed macaques (*M. nemestrina*), bearded pigs (*Sus barbatus*), and wild boars (*S. scrofa*) – in anthropogenic environments. Our evaluation identifies critical weaknesses in this study's analytical framework, including the pooling of data from two distinct macaque species, insufficient evidence to support the authors' claim of hyperabundance, and potential biases in statistical analyses. Based on a re‐analysis of the data using an adjusted statistical approach and drawing on the long‐standing body of research on macaques' behavioural ecology, we provide an alternative perspective on the challenges and threats faced by wildlife and humans in shared habitats.

## II. CRITICAL EVALUATION OF MOORE *ET AL.* (2023)

### (1) Macaque behavioural ecology

The selection of appropriate methodology is closely linked to a thorough understanding of the study species' behavioural ecology. This knowledge is essential for correctly interpreting results and drawing accurate conclusions about the taxa in question and their interactions with and impacts on ecosystems. Therefore, pooling species data for statistical analyses should be justified by shared traits, such as behavioural similarities, comparable ecological roles, or overlapping habitat requirements, rather than stemming from a lack of available data, as presented in Moore *et al*. (2023), who grouped *M. nemestrina* and *M. fascicularis* due to too ‘few […] observations’ (p. 1835) for each species if considered separately.

Although *M. fascicularis* and *M. nemestrina* share the same genus and are sympatric throughout much of the geographic range of *M. nemestrina* (Hansen *et al*., 2022; Ruppert *et al*., 2022), they exhibit some profound differences in their ecology and behaviour (Thierry, 2007a). These include variations in their degree of terrestriality (which is highly relevant for their detection success on near‐ground camera traps), dietary preferences, and adaptability to anthropogenic environments, which imply niche segregation (Rodman, 1991). The rather arboreal, largely frugivorous long‐tailed macaques typically thrive in wet alluvial terrain with thick ground cover and a continuous, dense canopy (Rodman, 1991). They preferentially inhabit forest edges near rural areas and human settlements, facilitated by their exceptional adaptability to anthropogenic habitats (Fooden, 1995; Gumert, 2011). By contrast, the elusive, predominantly terrestrial (Ruppert *et al*., 2018) southern pig‐tailed macaques are largely restricted to primary and secondary forests, preferring drier terrain on foothills and slopes (Rodman, 1991; Bersacola *et al*., 2019; Ruppert *et al*., 2022). Although they regularly forage in oil palm plantations, supplementing their omnivorous diet with a considerable amount of plantation rats (Ruppert *et al*., 2018; Holzner *et al*., 2019), these primates are rarely encountered in urban, human‐dominated areas (Ruppert *et al*., 2022).

Given these differences, the decision by Moore *et al*. (2023) to treat *M. fascicularis* and *M. nemestrina* as a single species in their analysis (p. 1835) raises concerns about the validity of their findings. This methodological approach is prone to overlooking key differences in species responses to human‐modified environments, potentially leading to misguided conservation strategies that fail to account for species‐specific ecological needs.

### (2) Locally abundant, but globally declining

Moore *et al*. (2023)'s conclusion on the presence of high densities of synanthropic species in anthropogenic habitats comes as no surprise. It is consistent with the long‐standing body of research on macaque behavioural ecology and their propensity for thriving in human‐altered habitats (e.g. Southwick & Cadigan, 1972; Fooden, 1995; Gumert, 2011; Hansen *et al*., 2019). However, applying the term ‘hyperabundant’ to a group of species that may exhibit high local densities in some areas while facing significant declines in their overall population sizes across their ranges (e.g. Luskin *et al*., 2017; Koch Liston *et al*., 2024) may strongly misrepresent the true conservation status of these species.

As correctly highlighted by Moore *et al*. (2023), three of the four study species are classified as either Endangered [*M. fascicularis* (Hansen *et al*., 2022); *M. nemestrina* (Ruppert *et al*., 2022)] or Vulnerable (*S. barbatus*; Luskin *et al*., 2017) in the IUCN *Red List*. Despite their ability to live at forest edges, forage in plantations, or scavenge in urban garbage bins, these synanthropes rely heavily on the presence of nearby forests and cannot thrive in agricultural or urban environments alone (Love *et al*., 2018; Tee *et al*., 2019; Holzner *et al*., 2021). This is especially concerning as cities expand and forest cover disappears (Global Forest Watch, 2023), significantly reducing suitable natural habitats for these species. Although alternative viewpoints exist based on the species' ability for population growth (Hilborn & Smith, 2023), recently published research clearly points towards a declining trend in the populations of *M. fascicularis* and *M. nemestrina* (see, e.g. Nuttall *et al*., 2022; Ruppert *et al*., 2022; Agger, 2023; Koch Liston *et al*., 2024). For example, a non‐invasive probability model developed by Koch Liston *et al*. (2024) revealed an 80% decline in the population of *M. fascicularis* over 38 years (1986–2022) across their range. Furthermore, Holzner *et al*. (2024) documented an exceptionally high mean annual infant mortality rate of approximately 60% over 10 years (2014–2023) in their study population of *M. nemestrina*, which inhabits a mixed forest–oil palm plantation habitat in Peninsular Malaysia, raising concerns about this species' long‐term viability in agricultural habitats.

Notably, Moore *et al*. (2023) did not demonstrate ‘hyperabundance’ according to their own definition of this term as ‘at least a doubling of [a species'] long‐term population density’ (p. 1831). The authors compare species densities and abundance across space and habitat types (i.e. degraded *versus* intact habitats) rather than assessing long‐term population trends in wild macaque and pig populations over time, despite the availability of relevant data.

**Fig. 1:**
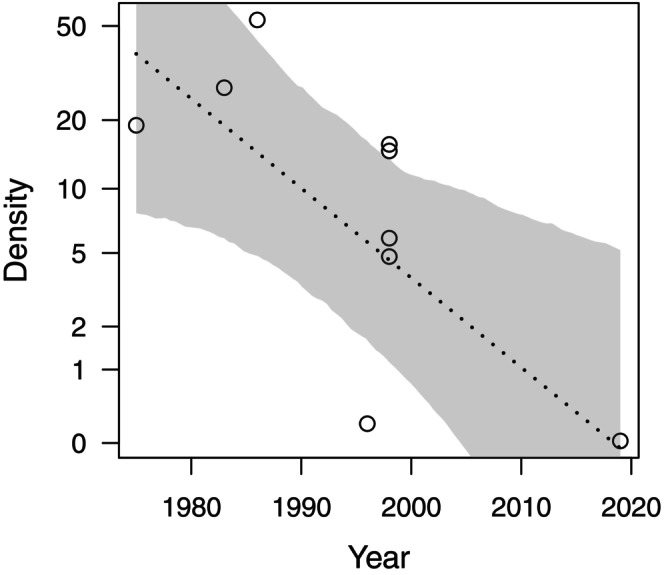
Population trend of *Macaca nemestrina*. Shown are density estimates (individuals/km^2^) as a function of time. The dotted line shows the fitted model and the shaded areas its 95% confidence intervals [*N* = 9 observations across six study sites; *y*‐axis is presented on a logarithmic scale; data set: Table S1 in Moore *et al*., 2023).

Using the data compiled by Moore *et al*. (2023) in their Table S1, including research spanning approximately 50 years, we modelled *M. nemestrina* density as a function of time. We fitted a linear mixed model (Appendix S1) with the sampling year as a test predictor and the study site as a random effect, given that the data may include multiple observations from the same area. Notably, our results showed a significant decline in the macaques' overall population density between 1975 and 2019 [model estimate ± standard error (SE) = −1.05 ± 0.42; likelihood ratio test (LRT): *χ* ^*2*^ = 5.74, *N* = 9, degrees of freedom (df) = 1, *P* = 0.017; Fig. 1].

Furthermore, Moore *et al*. (2023) acknowledge limitations in the available data but nevertheless claim that *M. fascicularis* density was 520% higher in degraded landscapes (31 individuals/km^2^) compared to intact forest habitats (5 individuals/km^2^, p. 1863). Crucially, this density estimate for intact forest, derived from a single survey conducted in Kelimutu National Park (Flores, Indonesia) in 2010 (Moore *et al*., 2023; Table S1), appears incorrect. The source cited (Fauzi *et al*., 2020) reports an *encounter rate* of ‘5 individuals/km’ (p. 5), which Moore *et al*. (2023) present as a density estimate. An encounter rate typically represents the number of individuals observed per distance of transect surveyed (e.g. individuals per km). By contrast, a population density estimate quantifies the number of individuals per unit area (e.g. individuals per km^2^). In fact, Fauzi *et al*. (2020) estimated population density of *M. fascicularis* only for a small, tourist‐frequented area within their study site, characterized by shrubs and locations of active food provisioning (p. 8: 104 individuals/km^2^). However, they did not provide a density estimate for the entire study site. Consequently, Moore *et al*. (2023)'s comparison of *M. fascicularis* density between degraded and intact forest habitats is unsupported, as no valid density estimate exists for intact forests. In this context, it is also noteworthy that Musser (1981) suggested that long‐tailed macaques have been introduced to Flores by humans, further raising concerns about the comparability of Fauzi *et al*. (2020)'s study population with native populations, given the potential for altered ecological dynamics resulting from the absence of natural predators.

### (3) Importance of forest integrity for predicting synanthrope relative abundance

Moore *et al*. (2023) used information on forest loss [estimated using the Forest Landscape Integrity Index (FLII), hereafter ‘forest integrity’; Grantham *et al*., 2020] and oil palm cover (estimated using the CRISP 2015 land cover map of Southeast Asia; Miettinen, Shi & Liew, 2016) based on the current characteristics of study sites and correlated these with population densities dating back to 1965 (see Table S1 in Moore *et al*., 2023). Given that forest loss and oil palm cover have increased rapidly over the past decades (Estoque *et al*., 2019), this method may incorrectly describe study areas as degraded with oil palm, when in fact, at the time of sampling, they still were intact forests. In particular, the authors' comparison of macaque and pig population densities between degraded and intact habitats may be flawed, as approximately 80% of the reported density estimates were obtained before 2010 (Table S1 in Moore *et al*., 2023).

Furthermore, it appears that the authors used 20 distinct statistical models on the same data set (Tables S2 and S3 in Moore *et al*., 2023), all aimed at addressing a single research question: the significance of anthropogenic habitat in predicting the relative abundance of synanthropes. This approach, which entails separate analyses for each species and predictor variable, critically inflates the Type I error rate (i.e. the risk of false positive conclusions; Andrade, 2019) while excluding potential interactions between predictors. For example, the impact of oil palm cover on species abundance might be positive only if the surrounding habitat is primarily characterized by intact forest (Love *et al*., 2018). However, this type of interactive effect was not adequately addressed by Moore *et al*. (2023).

Given the importance of considering interaction terms in regression analyses to enhance explanatory power and ensure accurate conclusions (Friedrich, 1982; Cohen *et al*., 2013), we suggest a re‐analysis of Moore *et al*. (2023)'s data set (their Tables S2 and S3) on camera trap estimates from studies published between 1993 and 2021. Accordingly, we constructed a single linear mixed model incorporating data from all four study species (Appendix S1), using the relative abundance index (RAI), that is the number of detections per 100 trap days, as the response variable. We considered forest integrity (mean ± SD = 4.88 ± 3.35, range = 0–9.75) and oil palm cover (low: 0–1% *versus* high: 21–42%) as fixed effects test predictors, species as fixed effects control predictor, and the study site as a random effect. We included the interaction between forest integrity and oil palm cover (as explained above), as well as these predictors' interactions with species, accounting for potential differences in species' responses to habitat disturbance.

**Fig. 2:**
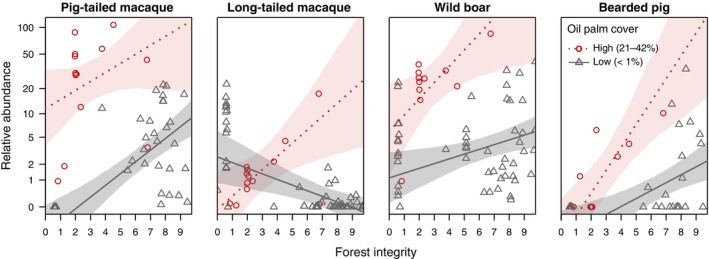
Impact of habitat degradation on species abundance. Relative abundance estimates (RAIs) as a function of forest integrity, shown separately for habitats with high (21–42%) and low (0–1%) oil palm cover, and for the four species. The lines show the fitted model and the shaded areas its 95% confidence intervals (*N* = 231 observations across 38 study sites; the *y*‐axis is presented on a logarithmic scale; data set: Tables S2 and S3 in Moore *et al*., 2023).

Overall, our results indicate a clear effect of the two test predictors on species' relative abundance (full‐null model comparison: *χ* ^2^ = 156.9, *N* = 231, df = 12, *P* < 0.001). Specifically, the significant three‐way interaction between forest integrity, oil palm cover, and species (LRT: *χ* ^2^ = 13.76, *N* = 231, df = 3, *P* = 0.003) underscores the particular importance of high forest integrity in mixed habitats where oil palm coverage ranges between approximately 20% and 40% (Fig. 2). This is in line with previous studies highlighting that although *S. barbatus* and *M. nemestrina* may regularly range in oil palm plantations, they depend on adjacent intact forests for shelter and to perform their full behavioural repertoire (Love *et al*., 2018; Holzner *et al*., 2021). A positive trend of forest integrity on relative abundance estimates can also be seen in habitats with low oil palm cover. An exception to this trend appears to be *M. fascicularis*, with this species entirely absent in 44% of sites with medium or high forest integrity (FLII >6; Grantham *et al*., 2020; Fig. 2). Their absence may indicate that this species has been extirpated from many forests and now persists at high densities only locally and in highly anthropogenic areas. Alternatively, this pattern may reflect a detection bias related to the data‐collection method using near‐ground camera traps. Some studies report that long‐tailed macaques tend to travel arboreally in intact forests (e.g. Cant, 1988; Rodman, 1991), which may reduce their detectability by terrestrial camera traps. By contrast, in more open habitats, such as agricultural or urban areas, the macaques' more terrestrial lifestyle, stemming from disruption of canopy connectivity, may increase their likelihood of detection, which may explain the higher detection rates of *M. fascicularis* in oil palm plantations and other degraded landscapes.

### (4) Potential threats at the human–wildlife interface

Moore *et al*. (2023) emphasize the ‘threats’ posed by synanthropes (macaques and pigs, in this case) ‘to both humans and nature’. However, rather than attributing these threats to the success of generalist species in human‐altered environments, we should focus on addressing the underlying causes: human activities that actively and rapidly create interfaces where wildlife is forced to adapt to modified landscapes, thereby increasing the risk of close interactions and disease transmission between humans and wildlife (Jones‐Engel, Schillact & Engel, 2003).

Moore *et al*. (2023) claim that synanthropes adversely affect forest ecosystems and displace other mammals through asymmetric competition. While these assertions may hold some truth (see, e.g. Bueno *et al*., 2011; Luncz *et al*., 2017; Cuevas *et al*., 2020), the authors fail to provide empirical evidence to support them, e.g. through seed germination experiments or assessments of habitat carrying capacity. Instead, they present a one‐sided perspective that overlooks the valuable ecological roles of certain synanthropic species in intact and degraded habitats. For instance, previous studies have highlighted the importance of macaques as seed dispersers, particularly in areas where other large vertebrates have been eliminated (Lucas & Corlett, 1998; Ruppert, Mansor & Anuar, 2014). Furthermore, the authors did not investigate instances of competitive exclusion. Without examining causal linkages, the observed association between the percentage of oil palm cover and the dominance of mammalian communities by southern pig‐tailed macaques and wild boars does not provide evidence of interspecific competition among mammals, nor does it implicate these species in cascading impacts. A more plausible explanation for the reduced mammal diversity in oil palm‐dominated landscapes is that the loss of intact forest habitat is more detrimental to mammalian taxa other than macaques and pigs. Previous research has shown that larger mammals and more specialized species, in particular, often struggle to adapt to highly degraded and anthropogenically impacted areas (Danielsen & Heegaard, 1995; Brodie, Giordano & Ambu, 2015; Alroy, 2017). Therefore, their displacement is likely a consequence of habitat conversion that disrupts ecosystem balance rather than a result of direct competition with more adaptable, generalist species.

As described by Moore *et al*. (2023), the presence of wildlife at the interfaces between natural habitats and rural or urban environments poses risks of transferring diseases, such as Nipah, monkeypox, or malaria, to humans. However, it is important to note that zoonotic diseases often spread in situations where humans directly handle animals, and cases of disease transmission associated with macaque capture, trade, and laboratory research are well documented (e.g. Greatorex *et al*., 2016; Hicks, 2023; Warne, Moloney & Chaber, 2023; Badihi *et al*., 2024; Linder *et al*., 2024). Conversely, evidence of disease transfer in the wild and in intact habitat settings is rare (e.g. Law, 2018). In fact, we would even argue that wildlife can contribute to mitigating human disease risks in shared environments by acting as a buffer. Research suggests that pathogen vectors, such as mosquitoes and ticks, may preferentially feed on macaques rather than humans in shared habitats (Lee *et al*., 2011), potentially reducing the risk of vector‐borne infections, such as malaria, in human populations.

While Moore *et al*. (2023) portray synanthropes as largely resilient, they are, in fact, exposed to severe anthropogenic threats. One of the key reasons for the long‐tailed macaques' recent classification as ‘Endangered’ by the IUCN is the intense hunting pressure and capture for the pet trade (Lappan & Ruppert, 2019; Hansen *et al*., 2021; Badihi *et al*., 2024), entertainment (SMACC, 2023), and biomedical and pharmaceutical research (e.g. Hansen *et al*., 2022; Nijman *et al*., 2022; Gamalo *et al*., 2023; Warne *et al*., 2023, Garber *et al*., 2024), starkly contradicting Moore *et al*. (2023)'s statement that macaques are ‘rarely hunted’ (pp. 1832). Further, in contrast to the authors' claim of ‘high fecundity’ (Moore *et al*., 2023, p. 1838), macaques are unequivocally classified as *K*‐selected species, whose life histories are characterized by larger body size, long lifespans, and the production of a limited number of offspring at a time (Ross, 1992). Specifically, macaques can live up to 28 years in the wild and typically produce single offspring at approximately two‐year intervals (van Noordwijk & van Schaik, 1999; Sponsel, Ruttanadakul & Natadecha‐Sponsel, 2002). Given their long lifespans and the complexity of source–sink population dynamics, the impact of other threats, such as chemical pollution often associated with agricultural intensification, may not be immediately apparent but could evolve into a more significant concern over the course of several generations. Holzner *et al*. (2024) provide initial indications that reproductive success is significantly reduced in a population of southern pig‐tailed macaques foraging in oil palm plantation areas, despite these macaques seemingly ‘thriving’ in agricultural landscapes.

Pigs, renowned for their typically higher fecundity than macaques, also encounter significant threats. Both bearded pig and wild boar populations have suffered from pronounced population declines due to recent outbreaks of African Swine Fever, posing imminent risks of extirpation across Southeast Asia (Luskin *et al*., 2021, 2023).

## III. CONCLUSIONS

(1) We emphasize the need for maintaining scientific integrity when assessing the population status of wildlife that appears to thrive in highly anthropogenic landscapes.
(2) We urge caution to avoid misrepresenting the actual dynamics of human–wildlife–habitat interfaces, as well as negative and sensational discourses, as this hampers the conservation management of these threatened species (Hansen *et al*., 2021). Concerningly, Moore *et al*. (2023)'s erroneous conclusions have been highlighted in global media (e.g. https://scitechdaily.com/they-were-everywhere-exploding-monkey-and-pig-populations-pose-human-disease-risk/#google_vignette) and actively used by the National Association for Biomedical Research to question the most recent IUCN Red List assessment of the endangered *M. fascicularis*.
(3) Rather than condemning species for their generalist nature and synanthropic capacities, it is essential to recognize human activity as the primary driver of habitat degradation that forces animals into anthropogenic areas and creates novel habitats for these species.
(4) The accurate and replicable assessment of available data, as well as the collection of new data on population sizes, distributions, and histories, is critical for effective scientific engagement and public discourse regarding synanthropic species in the Anthropocene.

## Supporting information


**Appendix S1.** Supplementary methods.
