# Supplementary material for: Threatened synanthropes depend on intact forests: a critical evaluation of Moore et al. (2023)
Source: Biol Rev Camb Philos Soc. 2025 Mar 11;100(4):1444–51. doi: 10.1111/brv.70007 (PMC12227786; doi:10.1111/brv.70007)
Supplement: Supplementary file 1 — Appendix S1. Supplementary methods. [file BRV-100-1444-s001.pdf]

Appendix S1. Supplementary methods

Statistical analysis

Linear Mixed Models (LMMs; Baayen, 2008) assessing the effects of our test predictors on southern pig-tailed macaque density (model 1) and the relative abundance of macaques and pigs (model 2) were implemented using the function *lmer* of the package 'lme4' (version 1.1.34; Bates *et al.*, 2015) in R (version 4.3.1; R Core Team, 2023).

LMMs were constructed based on the data sets presented by Moore *et al.* (2023). For model 1, we extracted all density estimates and their corresponding sampling years for southern pig-tailed macaques provided in their Table S1, yielding a sample size of nine observations from six study sites. For model 2, we combined information from their Tables S2 and S3, containing measures for oil palm cover and forest integrity, respectively. This allowed us to assign values of both predictor variables to each abundance estimate. Data points lacking information for either of the two covariates were excluded from the analysis, resulting in a sample size of 231 observations from 38 study sites.

To meet the assumptions of normally distributed and homogenous residuals, we log-transformed the response variables of both models. Verification of these assumptions involved visually inspecting a QQ-plot of the residuals (Field, 2013) and a scatterplot depicting the residuals plotted against the fitted values (Quinn & Keough, 2002). We added 1 to each data point of the response variable prior to the logarithmic transformation to account for zero values. Furthermore, we scaled and centred all continuous predictor variables to enhance model interpretation and convergence (Schielzeth, 2010). For model 2, we assessed collinearity between the predictors by determining variance inflation factors (VIFs) for a standard linear model lacking the interactions and the random effect using the function *vif* of the package 'car' (version 3.1.1; Fox & Weisberg, 2019). Based on this assessment, collinearity appeared to be no issue (maximum VIF: 1.06; Quinn & Keough, 2002).

In model 1, we examined the effect of the sampling year on southern pig-tailed macaque density. To do so, we compared the full model with a reduced model that lacked only our test predictor (i.e. the sampling year) but was otherwise identical to the full model using a likelihood ratio test (LRT; Barr *et al.*, 2013). In model 2, we evaluated the effects of forest integrity and oil palm cover on the relative abundance of macaques and pigs. In an overall test, we conducted a full-null model comparison based on a LRT (Dobson, 2001), whereby the null model lacked the two test predictors (i.e. forest integrity and oil palm cover) as well as their interactions. This approach aimed to avoid bias associated with multiple testing (Forstmeier & Schielzeth, 2011). Subsequently, we specifically tested the significance of the three-way interaction by comparing the full model with a reduced model lacking only the three-way interaction using a LRT (Barr *et al.*, 2013).

Limitations

Relative abundance indices (RAIs) derived from camera trapping data are widely used to estimate population abundance (O'Brien, 2011). However, caution is needed when interpreting these estimates, as they rely on the assumption that our ability to detect animals through camera traps is consistent across time, space, and species (Sollmann *et al.*, 2013). The overall activity and/or terrestriality of primates, for example, may decrease during prolonged rainfall in the monsoon season (Takemoto, 2004; Hanya *et al.*, 2018), potentially leading to a reduced detection probability through camera traps, which are typically placed at ground level.

Moore *et al.* (2023) attempted to account for differences in detection probabilities by incorporating N-mixture models into their analyses. However, this approach proved to be unsuitable for group-living animals, as it violates the assumption of regular N-mixture models that all individuals in the population are detected independently (Royle, 2004; Martin *et al.*, 2011).
